# Enhancing Prosthetic Workflow With Prototype Constructions in Dental Implant Treatment

**DOI:** 10.7759/cureus.97460

**Published:** 2025-11-21

**Authors:** Aleksandar Naydenov, Krasimir Chapanov

**Affiliations:** 1 Department of Prosthetic Dental Medicine, Medical University of Sofia, Sofia, BGR; 2 Department of Dental Implantology, Medical University of Sofia, Sofia, BGR

**Keywords:** 3d-printed, cad/cam, dental implantology, digital dentistry, prototype

## Abstract

This case report presents the concept of fabricating a 3D-printed prototype by outlining the benefits of these preliminary prosthetic constructions during implant treatment in a totally edentulous arch. Our study indicates that the final prosthetic construction anchored over dental implants demonstrates enhanced prosthetic features. Despite the advancements in digital dentistry, which offer significant diagnostic and planning capabilities, the fabrication of the final prosthetic construction remains a challenge even for specialists. This clinical case aims to present an in-house method for producing a 3D-printed prototype of the final prosthetic restoration, thereby minimizing clinical and laboratory errors such as incorrect selection of tooth form, size, and shade, improper occlusal contacts and articulation, suboptimal emergence profiles, and inadequate bridge design.

## Introduction

Implant‑supported full‑arch prostheses are a well-known option for rehabilitating edentulous patients, showing high survival and low complication rates [1]. The All‑on‑X concept (4-6 implants) provides a predictable solution for reduced bone volume in both jaws; however, mechanical complications, such as framework fracture, ceramic/composite chipping, or abutment/implant platform issues, may compromise patient satisfaction [2]. To minimize biological and mechanical complications during the early stages of treatment, the restoration must be rigidly fixed to the implants with balanced occlusion and light incisal guidance. An important factor to consider is the type and condition of the antagonist dentition (natural dentition, removable, or fixed denture) [3].

This report details a digital protocol using a prototype stage to transfer the planned outcome to the patient’s mouth for direct clinical evaluation and adjustment before definitive fabrication.

## Case presentation

A 50‑year‑old woman was referred to our clinic for full‑arch rehabilitation of the upper jaw. The patient was in good general health with no contraindications to implant therapy and was classified as ASA I, indicating a normal, healthy individual with no systemic disease. She had been edentulous in the maxilla for more than two years, and reported poor function and speech due to the mobility of a conventional complete denture fabricated by a general dentist.

A cone‑beam computed tomography (CBCT) scan, photo documentation, and digital impressions were obtained. Optical impressions were captured with an intraoral scanner (Virtuo Vivo™, Dental Wings/Institut Straumann AG, Montreal, Canada). Based on the clinical and radiographic examination, no active pathology was present. Bone deficiency was noted in the distal regions of the maxilla, with adequate bone volume and quality in the zone between teeth 15-25. Risk assessment classified the case as low risk [4].

Several treatment plans were proposed. The patient consented to the placement of six implants (GM Helix®, Neodent) following a Type 4 protocol, which involves late placement in a fully healed soft and hard tissue site with immediate loading [5].

Digital planning

**Figure 1:**
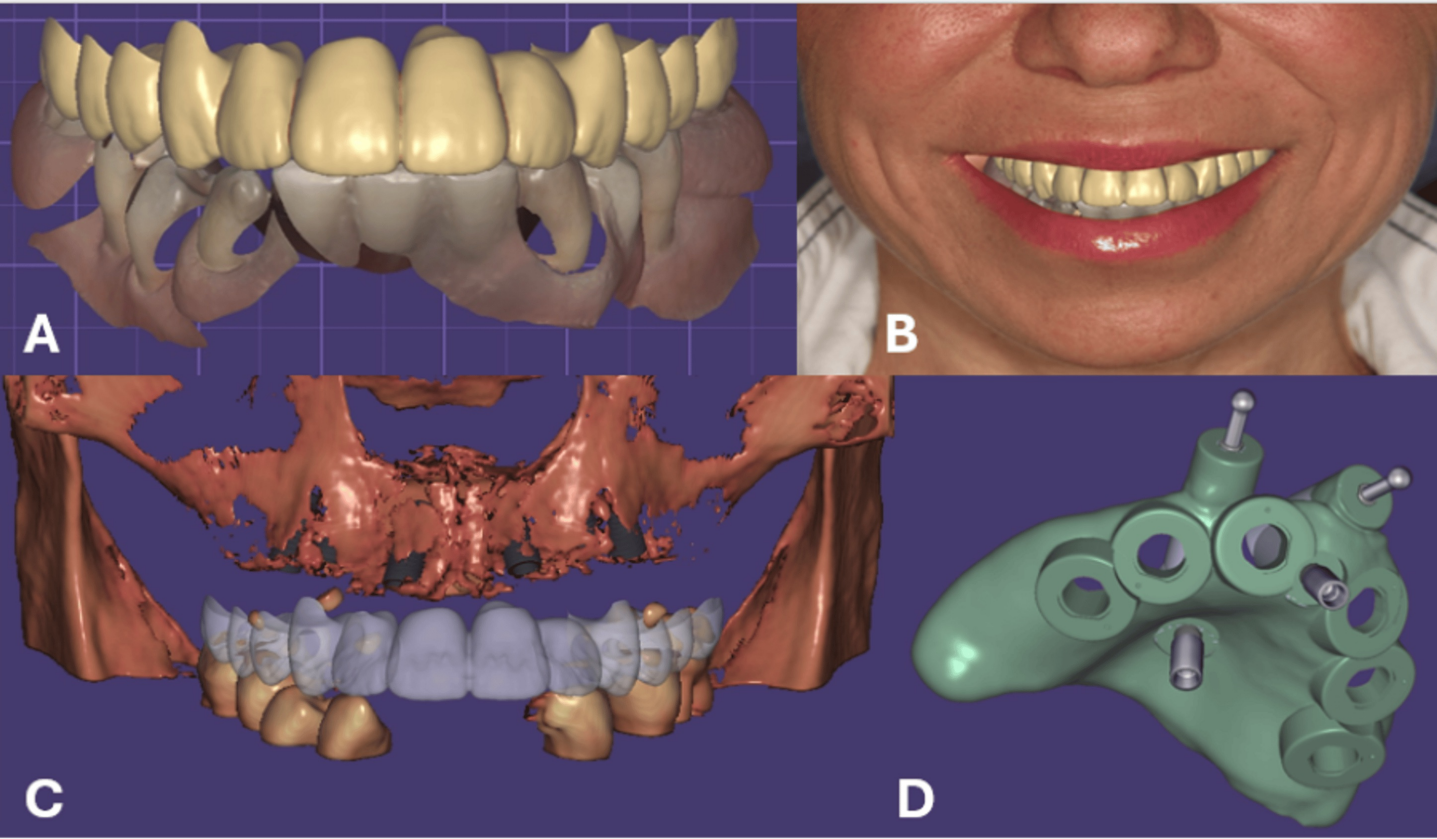
A) Digital patient image was created in “ExoCad” software; B) Digital smile design created after combining information from the extraoral photo. Digital impression derived from intraoral optica and CBCT; C) Virtual position of the implants according to CBCT and DSD; D) Design of the surgical guide.

A virtual patient was created in ExoCad® (ExoCad GmbH, Germany) by integrating 2D facial photographs, intraoral scans, and CBCT data. Occlusal vertical dimension (OVD) and the reproducible physiological position of the lower jaw (RPPLJ) were registered with the aid of the existing denture. After the wax-up and smile design were approved, a surgical guide was designed and fabricated. The virtual patient, smile design, and the surgical guide design are presented in Figure 1.

**Figure 2:**
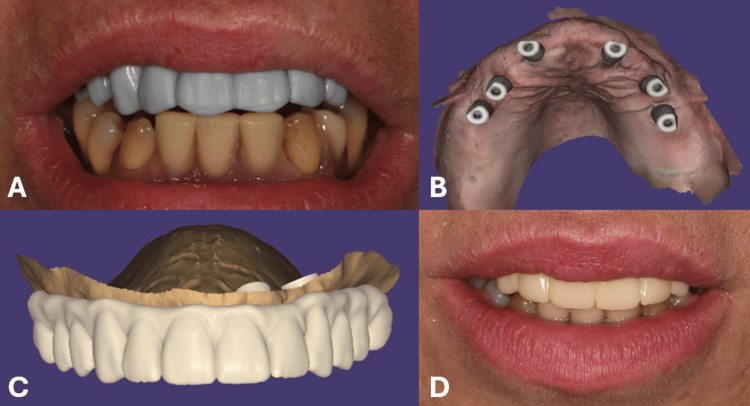
A) Intraoral adaptation of the 3D printed prototype 1; B) Digital impression after placement of the implants; C) Digital design of the provisional immediate prosthetic construction; D) The provisional prosthetic construction delivered immediately after placement of the implant.

The virtually planned implant positions were verified intraorally prior to the procedure using resin-printed Prototype 1 (Aqua 8K Resin, Phrozen, Taiwan), which had been fabricated in advance of the procedure (Figure 2). Local anesthesia was administered, after which the surgical guide was secured with anchoring pins. Using computer-guided implantology, six bone-level implants (Acqua, GM Helix®, Neodent, Brazil) were placed in the maxilla without raising a mucoperiosteal flap. The implants were 4 mm in diameter and 10 mm in length, except those in the region of teeth 16/26, where implants of 4.3 mm in diameter and 10mm in length were used. All implants achieved high insertion torque and adequate primary stability, thus enabling immediate restoration and loading. After confirming that the implant positions matched the virtual plan, scan bodies were attached, and an intraoral scan was performed (Figure 2), after which six mini conical abutments were installed.

The intraoral adaptation of Prototype 1, the post-placement digital impression, the design of the immediate prosthesis, and its delivery are shown in Figure 2.

When the surgical procedure was completed, the prefabricated provisional prosthesis (fabricated in the dental laboratory) was screw-retained intraorally without the need for additional intraoral relining or contouring materials. The screw-access channels were filled with PTFE tape (polytetrafluoroethylene - "Teflon") and sealed with light-cured composite resin. Occlusal contacts were verified, and minor adjustments were made. The postoperative instructions were explained to the patient.

**Figure 3:**
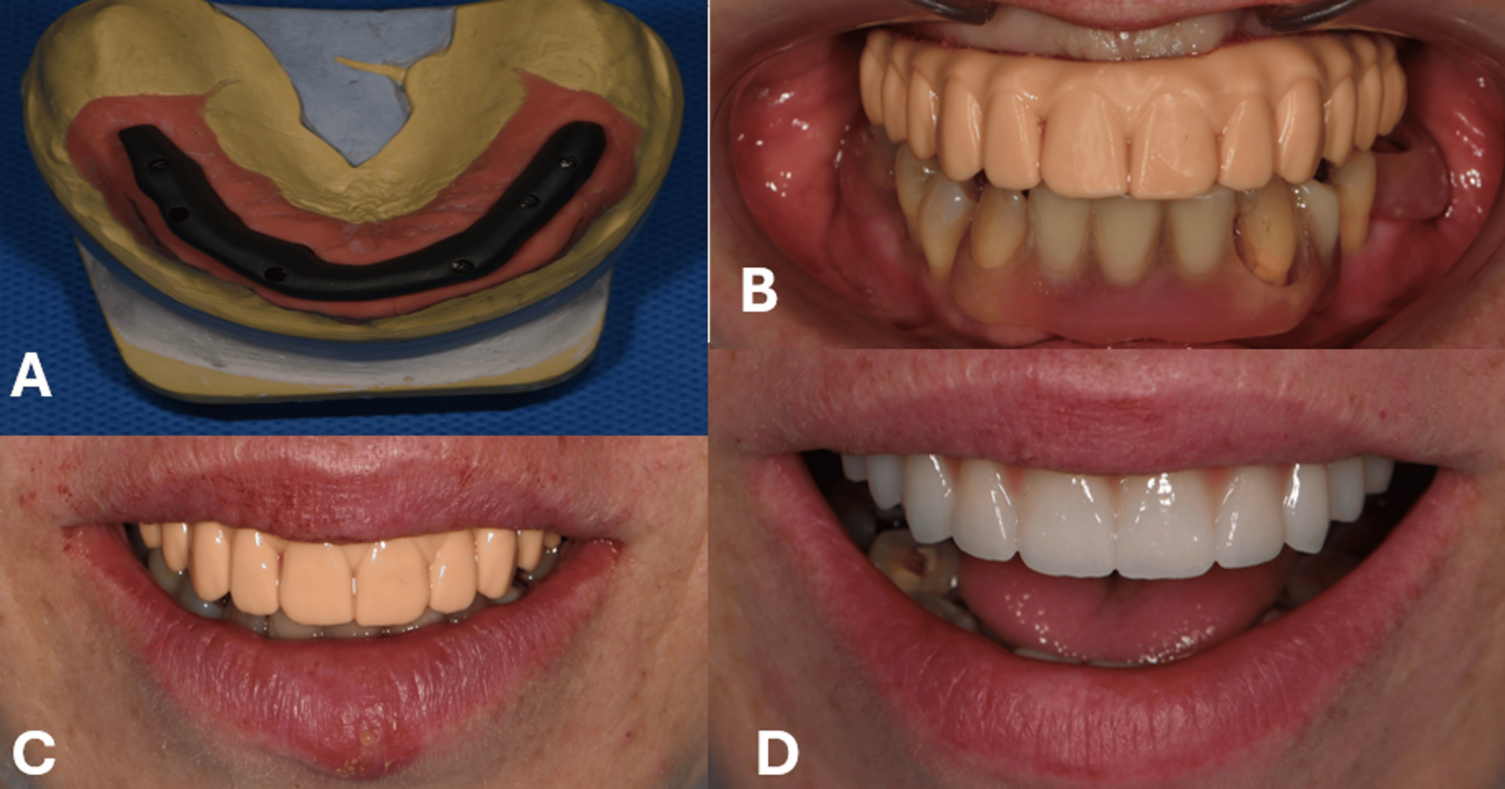
A) Titanium milled bar at the stone model; B) Intraoral adaptation of the Prototype 2; C) Extraoral verification of Prototype 2 after adjustments were made; D) Construction of the final prosthesis.

Six months later, a conventional A-silicone impression (Variotime®, Kulzer GmbH, Germany) was taken at the abutment level and sent to the laboratory, where the provisional prosthesis was scanned. A titanium bar was milled for the screw-retained FP2 hybrid prosthesis [6]. Figure 3 illustrates the titanium bar, intraoral and extraoral verification of Prototype 2, and the final prosthesis.

Optimization of the prosthesis was implemented at this stage. The final prosthesis, to be milled from zirconia (Katana™ Zirconia, Kuraray Noritake Dental Inc., Japan), was first 3D-printed (Aqua 8K Resin, Phrozen, Taiwan) as Prototype 2. The prototype was used to verify functional planes, occlusal contacts, and tooth morphology. After patient approval, the final zirconia construction was fabricated and screw-retained onto the implants. No adjustments were required at the time of delivery (Figure 3). The screw-access channels were filled with PTFE (polytetrafluoroethylene) and sealed with light-cured composite resin.

## Discussion

The adopted digital approach enables precise planning and safe surgical interventions, simplifying clinical steps and enhancing communication between dental specialists and patients [7]. Advancements in devices for acquiring digital impressions (e.g., intraoral scanners) have revolutionized the workflow by delivering high accuracy in both planning and execution while ensuring patient comfort [8]. Protocol type 4, which involves implantation in a fully healed site (more than 6 months after extraction), was chosen because the patient was referred for implant treatment too late after the extractions.

CAD software plays a crucial role in creating a “virtual patient” model, enabling treatment planning tailored to functional and aesthetic requirements [9]. The virtual-patient concept integrates data from facial and intraoral scans, digital setups or wax-ups, CBCT imaging, and photographic documentation into a unified case. This facilitates planning of ideal implant positions, visualization of the final tooth arrangement, and design of the prosthetic restorations. Additionally, CAD tools support analysis of the collected data, thus providing options for smile design and predictive outcome assessment [10].

Traditionally, implant-supported full-arch prosthetic restorations are screw-retained. However, cement-retained restorations offer aesthetic advantages, particularly in cases of implant angulation discrepancies. Efforts to combine the benefits of screw-retained and cement-retained designs have led to hybrid techniques, often involving a screw-retained metal (typically titanium) mesostructure paired with a cemented zirconia or polymeric superstructure [11].

Challenges in fabricating hybrid prostheses may arise during both clinical and laboratory stages. The expertise of clinicians and technicians, combined with advanced planning software, minimizes errors when rehabilitating fully edentulous patients. Collaboration with skilled dental technicians reduces laboratory errors such as inaccuracies in master-model casting, gingival-mask fabrication, and digitization. Proficiency with software tools for designing and manufacturing meso- and superstructures further ensures precision. A rigorous laboratory workflow during production of the final hybrid construction supports excellent aesthetic, phonetic, and functional outcomes while minimizing biological and mechanical complications.

While software-driven algorithms facilitate smile design, successful clinical translation requires a multidisciplinary approach. To enhance precision and reduce errors, a prototyping stage is often warranted, which includes 3D printing the intended prosthetic superstructure. Such prototypes enable verification of tooth shape and size, evaluation of smile lines, reference planes, occlusal contacts, and articulation. Necessary corrections can then be made to the virtual-patient model before final production. The use of 3D-printed prototypes streamlines and accelerates treatment by allowing the digitally planned result to be transferred directly to the patient’s oral cavity for clinical evaluation and adjustment as needed.

## Conclusions

The use of 3D-printed prototypes as an intermediate step enhances the accurate selection of artificial teeth shape and size, and smile line evaluation. It also allows for verification of the occlusal plane and contacts. Corrections made to the prototype can then be transferred to the virtual patient model, allowing the prosthetic design to be finalized. The application of 3D-printed prototypes significantly streamlines treatment by facilitating the intraoral transfer of digital planning, thereby enabling direct clinical assessment and adjustment when required.

## References

[1] Rutkowski JL (2022). Survival rates of dental implants versus teeth. J Oral Implantol.

[2] Soto-Penaloza D, Zaragozí-Alonso R, Penarrocha-Diago M, Penarrocha-Diago M (2017). The all-on-four treatment concept: systematic review. J Clin Exp Dent.

[3] Penarrocha-Diago M, Penarrocha-Diago M, Zaragozí-Alonso R, Soto-Penaloza D, On Behalf Of The Ticare Consensus M (2017). Consensus statements and clinical recommendations on treatment indications, surgical procedures, prosthetic protocols and complications following All-On-4 standard treatment. 9th Mozo-Grau Ticare Conference in Quintanilla, Spain. J Clin Exp Dent.

[4] 4] “Anthony Dawson / William C. Martin / Waldemar D (2024). The SAC Classification in Implant Dentistry,” Quintessence Publishing Company, Inc. Accessed: Dec. 11. https://www.quintessence-publishing.com/usa/en/product/the-sac-classification-in-implant-dentistry#qpproductdigitaltab.

[5] Gallucci GO, Hamilton A, Zhou W, Buser D, Chen S (2018). Implant placement and loading protocols in partially edentulous patients: a systematic review. Clin Oral Implants Res.

[6] Misch Misch, Carl E. (2014). Dental Implant Prosthetics. https://shop.elsevier.com/books/dental-implant-prosthetics/misch/978-0-323-07845-0.

[7] da Silva Salomão GV, Chun EP, Panegaci RD, Santos FT (2021). Analysis of digital workflow in implantology. Case Rep Dent.

[8] Michelinakis G, Apostolakis D, Tsagarakis A, Kourakis G, Pavlakis E (2020). A comparison of accuracy of 3 intraoral scanners: a single-blinded in vitro study. J Prosthet Dent.

[9] Joda T, Gallucci GO (2015). The virtual patient in dental medicine. Clin Oral Implants Res.

[10] Joda T, Zarone F, Ferrari M (2017). The complete digital workflow in fixed prosthodontics: a systematic review. BMC Oral Health.

[11] Scarano A, Stoppaccioli M, Casolino T (2019). Zirconia crowns cemented on titanium bars using CAD/CAM: a five-year follow-up prospective clinical study of 9 patients. BMC Oral Health.

